# Rhus Texicodendron—The Cause of Milk Sickness

**Published:** 1861-09

**Authors:** E. C. Landrum

**Affiliations:** Columbus, Mississippi


### Medical and Surgical Journal.

VOL. VIIA. SEPTEMBER, 1861. [NO. 1.

### ORIGINAL COMMUNICATIONS.

### ARTICLE I.

*Rhus Toxico-dendron—The Cause of Milk Sickness—A review of an Article on this Subject by S. C. Chase, M. D., Harmar, Ohio—first published in the Lancet & Observer, and re-published in the Atlanta Medical and Surgical Journal.* By Z. C. LANDRUM, M. D., Columbus, Mississippi.

Dr. Chase in his communication states: "I find in the March number of your journal an address on the above subject, by Isaac Mendenhall, M. D., of Ashland, Ind. Nor was I aware, until I saw his essay, that the cause of this Malady was unknown, generally, to the medical faculty. I have been a practitioner fourteen years, during all of which time I have regarded the cause of Milk sickness as easily traceable to its origin as the cause of ague, or any of the endemics, with this difference, that while the cause of endemics usually depends upon atmospheric conditions, the presence of miasmata, etc., the cause of Milk Sickness depends upon the presence of vegetation, which abounds in certain localities only, for which reason the cattle of fallow ground pasture fields are exempt, while those of the adjacent wood pasture are trembling with the disease. In such wood pasture will be found the Rhus Toxicodendron growing every where among the grass, so that if the cattle eat grass they must also eat Rhus. Notwithstanding, instinct enables dumb animals to avoid some poisonous plants, yet in dry seasons cattle will devour the Rhus with avidity, and have no objections to the foliage of the buck-eye, the latter of which often produces as serious consequences (as far as the animal is concerned) as the former."

The Doctor proceeds to state that during the prevalence of this disease in 1841-42, in Marion County, Ohio, a Dr. Brown and himself commenced an investigation of its cause. They ascertained that cattle pastured on enclosed, cultivated fields, where the Rhus did not exist, were exempt from the disease, whilst those turned out in wood pastures, and open prairie, in which there was profuse growth of this poisonous vegetation intermixed with the grass, suffered. As the cause was not atmospheric, and did not exist in the water, the drink being the same in both cases, they concluded that it must have been and was the Rhus. They state further that the premises were carefully examined, and no other poisonous vegetable was found. Again, he says: "So poisonous is Rhus that it pollutes the atmosphere where it grows, so that children, as well as some grown persons, who go among it to gather berries from bushes growing in the same locality are badly poisoned."

We are satisfied that these extracts contain a number of gross errors, we therefore, purpose to furnish briefly in this communication facts which refute them. In the counties of Lowndes and Noxubee, Miss., all of the good grazing lands, such as existed in the open prairies previous to the settlement of the country, are now in cultivation. These lands are very rich, have been reduced to a fine state of culture, and are annually planted in corn and cotton. They were once covered with the finest grass, waiving in summer verdure from waist to head high, and supplied thousands of wild deer and cattle with grazing. This pasturage having been destroyed, there is none now left, at a distance from the Bigbee river, but the open woods and sloughs. The woods contain no grass, and the sloughs but very little.

These wide lands are settled by wealthy farmers who own large numbers of negroes, and have no more open cleared land than is needed for corn and cotton. Therefore this is not a stock-raising country. Indeed the culture of cotton is so much more profitable, that the raising of grasses and stock would be neglected, even if lands were abundant for this purpose. But few farmers raise or keep more cattle than are sufficient to make their milk and butter. And to succeed in this, many are compelled, on account of the scarcity of grasses, to feed their cows both winter and summer. In the woods and sloughs where the cattle find their scanty pasturage there is such a profusion of Rhus Toxico-dendron, and such luxuriance in its growth, as I have never seen elsewhere. In the sloughs on their winter creeks (for they are all dry in the summer season) where the cattle spend most of the day nipping buds, and weeds (there being but little grass on any of them, and none at all on many,) I think nearly a third of the trees of any size have twined about their bodies and around their limbs this poisonous vine, clustered thick with the most luxuriant green foliage. Indeed, it is difficult to ride or walk through the sloughs without being touched repeatedly with the leaves of the Rhus. The overseers on the plantations tell me that they have frequently seen the cattle bite off and eat the leaves of this vine as high as they can reach on the trees, and yet no such disease as Milk Sickness has ever prevailed in this country that I am aware of. If the Rhus were a cause of Milk Sickness, our cattle would certainly be affected. Dr. Chase admits that in dry seasons when grass is scarce, the instincts of cattle does not cause them to avoid Rhus, but on the contrary, "they will devour it with avidity." There is a scarcity of grass wherever cattle roam, both in dry and in wet seasons; there is an abundance of Rhus above any other species of plants on vines, poisonous or innocuous; and our cattle, in all probability, consume as much or more of this poisonous vine, on account of these two circumstances, than they do in any other country; yet we have no Milk Sickness among them. The facts are precisely as I have stated them; if so, can the eating of Rhus by cattle be a cause of Milk Sickness?

The statement that emanations from the Rhus so pollutes the atmosphere where it grows, that children and some grown persons are poisoned by it, we think utterly unfounded. No such results have ever been experienced in this country.

We have ourselves frequently been poisoned by this vine, and we have also witnessed in others all the severe effects of which Dr. Chase speaks; but we do not believe its poisonous properties are ordinarily imparted to the surface of the human body without bruising the leaves either by rubing or mashing. The atmosphere about this plant will not poison, nor do we believe that simple contact of the leaves or the vine with the surface for a short time without bruising or rubbing, will produce such a result. It is not uncommon at all for the leaves to strike us on the face and on the hands in riding through these sloughs; but such contact has never yet poisoned us. Moreover, we have chewed the leaves in our mouth without being poisoned. These statements are in striking contrast with those made in Chase's communications, and we are at a loss to account for such a wild difference in the results of our observation. Science is made up of facts gathered from the observation and study of different persons, and its beautiful proportions are hideously mared by any mixture of truth and error; and we felt therefore that the confident statements of Dr Chase about this obscure disease demanded refutation, before they were sealed with the stamp of truth.

